# Low Levels of Serum and Intracellular Vitamin C in Hospitalized COVID-19 Patients

**DOI:** 10.3390/nu15163653

**Published:** 2023-08-20

**Authors:** Lara S. Boerenkamp, Birgit L. M. G. Gijsbers, Erik-Jan Ververs, Eva M. S. Pijpers, Bart Spaetgens, Aniek de Coninck, Wilfred T. V. Germeraad, Will K. W. H. Wodzig, Lotte Wieten, Gwendolyn N. Y. van Gorkom, Catharina H. M. J. van Elssen

**Affiliations:** 1 Division of Hematology, Department of Internal Medicine, GROW School for Oncology and Developmental Biology, Maastricht University Medical Center, 6229 HX Maastricht, The Netherlands; 2 Department of Internal Medicine, Maastricht University Medical Center, 6229 HX Maastricht, The Netherlands; 3 Central Diagnostic Laboratory, Department of Clinical Chemistry, Maastricht University Medical Center, 6229 HX Maastricht, The Netherlands; 4 Department of Transplantation Immunology, Maastricht University Medical Center, 6229 HX Maastricht, The Netherlands

**Keywords:** vitamin C, COVID-19, immune cells

## Abstract

Vitamin C is a crucial micronutrient for human immune cell function and has potent antioxidant properties. It is hypothesized that vitamin C serum levels decline during infection. However, the precise mechanisms remain unknown. To gain deeper insights into the true role of vitamin C during infections, we aimed to evaluate the body’s vitamin C storage during a SARS-CoV-2 infection. In this single-center study, we examined serum and intracellular vitamin C levels in peripheral blood mononuclear cells (PBMCs) of 70 hospitalized COVID-19 patients on the first and fifth days of hospitalization. Also, clinical COVID-19 severity was evaluated at these timepoints. Our findings revealed a high prevalence of hypovitaminosis C and vitamin C deficiency in hospitalized COVID-19 patients (36% and 15%). Moreover, patients with severe or critical disease exhibited a higher prevalence of low serum vitamin C levels than those with moderate illness. Serum vitamin C levels had a weak negative correlation with clinical COVID-19 severity classification on the day of hospitalization; however, there was no correlation with intracellular vitamin C. Intracellular vitamin C levels were decreased in this cohort as compared to a healthy cohort and showed further decline during hospitalization, while serum levels showed no relevant change. Based on this observation, it can be suggested that the reduction of intracellular vitamin C may be attributed to its antioxidative function, the need for replenishing serum levels, or enhanced turnover by immune cells. These data give an incentive to further investigate the role of intracellular vitamin C in a larger and more heterogeneous cohort as well as the underlying mechanisms.

## 1. Introduction

Vitamin C is an important micronutrient for the human immune system. As an antioxidant, it plays an important role in reducing oxidative stress [1]. Moreover, vitamin C has an interesting role in the regulation of immune function, and various immune cells are affected by it [2,3]. It modulates the balance between pro-inflammatory and anti-inflammatory cytokines [4], resulting in increased chemotaxis, phagocytosis, and microbial killing by neutrophils [5,6]. Vitamin C accumulates in neutrophils, and a process called recycling takes place, creating high concentrations of vitamin C intracellularly and lower concentrations extracellularly. This effect creates more oxidant reduction inside the cells and more oxidants outside the cell for improved microbial killing [7]. Furthermore, by promoting T helper 1 (Th1) skewing, it facilitates the activation of cytotoxic T cells (CTL), and it has been proven to be an absolute prerequisite for T cell development [8,9]. In vitro studies have also demonstrated that vitamin C stimulation enhances the maturation and proliferation of natural killer (NK) cells [10,11]. Our findings, as well as those of others, have highlighted the accumulation of vitamin C in various immune cells, underscoring its pivotal role as an important micronutrient crucial for the development and optimal function of these cells [12,13]. Vitamin C has numerous additional functions beyond the ones described above; it serves as a cofactor or cosubstrate for various enzymes, for example, in collagen hydroxylation, which plays a role in wound healing, and in the production of noradrenalin [7].

In humans, the heavily mutated Gulo gene is nonfunctional due to evolutionary processes, and therefore humans are not able to synthesize vitamin C to maintain a normal serum level and hence depend on dietary intake and storage. Vitamin C uptake takes place in the intestine through the glucose transporter (GLUT) and the sodium-dependent vitamin C transporter (SVCT) 1. The SVCT1 receptor is also responsible for tissue uptake of vitamin C in epithelial cells, whereas the SVCT2 receptor is responsible for vitamin C uptake in more specialized cells of the adrenal gland, the brain, and lymphocytes [14]. Intra- and extracellular vitamin C levels have been shown not to correlate, and as opposed to serum vitamin C, intracellular levels, which can be up to 50–100 times higher than serum levels, do not show a dose-dependent increase [15]. Cells that express the SVCT2 receptor show a high intracellular vitamin C concentration, which protects these metabolically active cells from oxidative stress [16,17]. Additionally, vitamin C storage in these cells preserves their function during vitamin C depletion. Therefore, symptoms of vitamin C deficiency only occur when the intracellular resources are exhausted. This occurs, depending on the different tissues, days to weeks after serum vitamin C becomes within the deficient range [18]. As a result, it has been postulated that intracellular vitamin C concentrations are more reflective of the body’s accurate vitamin C status than serum vitamin C.

In the general healthy population, the occurrence of hypovitaminosis C is approximately 12% [19,20]. However, studies have revealed that hospitalized patients exhibit a prevalence nearly seven times higher [21]. Patients with infectious diseases are particularly susceptible to vitamin C deficiency [21,22]. Most prominent are the extremely low vitamin C levels in septic and critically ill patients admitted to the intensive care unit (ICU), even when receiving standardized nutrition [23]. In these patients, low vitamin C levels have been associated with severity of illness and poor outcome [24,25].

The decreased vitamin C levels observed during infectious diseases are most likely caused by an increased production of reactive oxygen species (ROS), resulting in more scavenging of ROS by vitamin C [22]. Vitamin C plays a crucial role in numerous antioxidant and anti-inflammatory processes [13], leading to an increased demand for vitamin C in patients suffering from infectious diseases. These well-established functions of vitamin C have prompted several clinical studies investigating the effect of vitamin C treatment on critically ill patients in the ICU. While results vary widely across the different studies, a comprehensive meta-analysis suggests the clinical benefit of administering an intermediate dose of intravenous vitamin C to septic ICU patients [26]. This clinical benefit was reflected by a decrease in the duration of vasopressor support and mechanical ventilation [27,28]. Additionally, other studies showed a reduction in ICU stays and lower mortality rates [26,29]. Variations in study outcomes may be attributed to differences in patient populations and the dosages of vitamin C administered. 

Several studies have examined the effect of vitamin C supplementation on the prevention and treatment of the common cold [30]. Similar to the ICU studies, conflicting results have been reported. However, while vitamin C supplementation does not affect the incidence of the common cold in the general population, the most consistent finding is a reduction in the duration and severity of cold symptoms when vitamin C is administered within 24 h after symptom onset and continued for at least 5 days [30]. It is thought that the beneficial effects are mostly explained by the antioxidant functions of vitamin C, thereby reducing further tissue damage, and by its immunomodulatory effects, which create a less pro-inflammatory environment [10]. Inspired by this compelling data, we hypothesized that vitamin C levels might also reduce symptoms following a SARS-CoV-2 infection. Severe or critical COVID-19 is often associated with a pro-inflammatory state or cytokine storm [31]. Vitamin C might be able to reduce the pro-inflammatory state through its immune-modulating and antioxidant functions. To investigate this, we set out to not only investigate serum vitamin C levels but also intracellular storage by analyzing intracellular vitamin C levels in leukocytes. Serum vitamin C levels are highly susceptible to acute changes, such as decreased dietary intake, and might therefore not accurately reflect the true vitamin C status [12,18]. In contrast, intracellular vitamin C levels in leukocytes represent vitamin C storage better as they are less sensitive to acute changes [12]. Leukocyte vitamin C levels are up to 30–100 times higher than plasma levels and offer a more accurate reflection of tissue levels [12,32]. In septic patients, Carr et al. showed normal intracellular vitamin C levels in neutrophils despite low serum vitamin C levels; however, these cells exhibited increased ex vivo uptake of vitamin C compared to neutrophils from healthy donors, which displayed negligible vitamin C uptake [33]. Intracellular vitamin C levels in immune cells may correlate more specifically with immune function than serum vitamin C levels, and changes in these levels may provide further insights into the use of vitamin C during infections. 

To the best of our knowledge, the assessment of intracellular vitamin C levels in leukocytes has not been explored in COVID-19 patients. Therefore, the objective of this study is to examine vitamin C levels in both serum and intracellular fluid during the hospitalization of COVID-19 patients. By doing so, we aim to gain valuable insights into the dynamics of vitamin C metabolism during infection and determine whether these levels correlate with disease progression and severity. 

## 2. Materials and Methods

### 2.1. Patients

This study was a single center prospective cross-sectional cohort study. It was conducted at the COVID-19 ward at the Maastricht University Medical Center (MUMC+). Inclusion of participants took place from 2 December 2020 until 10 March 2021 and from 13 December 2021 until 23 March 2022. The study was approved by the medical ethical committee at the MUMC, and all procedures were conducted in accordance with the ethical principles outlined in the Declaration of Helsinki, the ICH-GCP Guidelines, and the EU Clinical Trial Directive (2001/20/EG). Inclusion criteria consisted of the following: (1) symptomatic patients primarily admitted to the COVID-19 ward with a positive SARS-CoV-2 PCR; (2) an age of 18 years or older; and (3) the provision of written informed consent. Patients underwent nasopharyngeal swab polymerase chain reaction (PCR) testing for SARS-CoV-2. 

### 2.2. Collection of Clinical Data

The data were extracted from the electronic medical records. Baseline characteristics were collected on day 1 of their hospitalization. Additional outcome measures, such as the maximal clinical COVID-19 severity classification (according to Chinese Clinical Guidance for COVID-19 Pneumonia Diagnosis and Treatment, 7th edition [34]), ICU admission, duration of ICU stay, duration of mechanical ventilation, and time until discharge or death, were recorded. The definition of the clinical COVID-19 severity classification was as follows: mild (mild symptoms, no sign of pneumonia on chest imaging), moderate (fever and respiratory symptoms, chest imaging shows pneumonia), severe (dyspnea with a respiratory rate of 30 times/minute or higher, and/or oxygen saturation of 93% or lower, and/or alveolar oxygen partial pressure/fraction of inspiration O_2_ (PaO_2_/FiO_2_) of 300 mmHg or lower), and critically ill (respiratory failure needing mechanical ventilation, and/or shock, and/or organ failure in need of ICU monitoring and treatment; this included patients that met this definition but had an advance directive of no ICU admission). The sequential organ failure assessment (SOFA) score, C-reactive protein (CRP), and lactate dehydrogenase (LDH) are established clinical biomarkers that predict clinical COVID-19 severity and clinical outcome [26,35] and were therefore also collected. 

### 2.3. Collection and Analysis of Blood Samples

Blood samples were collected twice from each participant, once on day 1 of hospitalization and again on day 5 of hospitalization, with a time range of 72 h. Both serum and intracellular vitamin C levels were measured. For serum vitamin C level determination, a commercial kit from Chromsystems (Gräfelfing, Germany) was used. This analysis was performed by the central diagnostic laboratory of the MUMC+, which holds ISO 15189 accreditation for this routine measurement. The steps include protein precipitation and vitamin C stabilization, followed by quantification using a stable internal standard with high-performance liquid chromatography (HPLC) with UV detection. The measurement of the intracellular vitamin C concentration of peripheral blood mononuclear cells (PBMCs) was conducted after extracting vitamin C from the PBMCs, also using HPLC with UV detection. First, lymphoprep density centrifugation was used to separate PBMCs from 8 mL of heparinized blood. Then, a washing step and cell count took place; 2 × 10^6^ PBMCs were then resuspended in ammonium acetate, followed by the addition of precipitation reagent. The sample was thoroughly mixed and then incubated for 10 min. The supernatant was frozen at −80 °C until final analysis. More detailed information about this method has previously been described [36].

### 2.4. Data Analysis

Continuous variables were presented as mean ± standard deviation in the case of a normal distribution or median with interquartile range (IQR) in the case of a non-normal distribution. The Shapiro–Wilk test was used to assess normality. Categorical data were presented as counts with percentages. Frequencies were presented as percentages or proportions and compared to population numbers by performing a one-sample binomial test. To compare paired groups over time, paired *t*-tests were used, assuming a normal distribution of the variables. Unpaired t-tests were used to compare vitamin C levels in patients with improving disease versus patients with progressive disease. 

Spearman’s rank test was performed to examine the correlation between serum and intracellular vitamin C levels with clinical COVID-19 severity, SOFA score, duration of hospital stay, and duration of ICU admission when non-normally distributed variables were involved. Binary logistic regression was performed to assess the risk of low vitamin C in different patient populations based on the COVID-19 severity score. The significance level (α) was set at 0.05. Statistical analysis was performed using IBM SPSS Statistics for Windows, Version 27.0 (IBM Corp., released 2020; Armonk, NY, USA). For data visualization, GraphPad Prism version 9.4.1 for MacOS (GraphPad Software, San Diego, CA, USA) was used. 

## 3. Results

### 3.1. Inclusion

**Figure 1:**
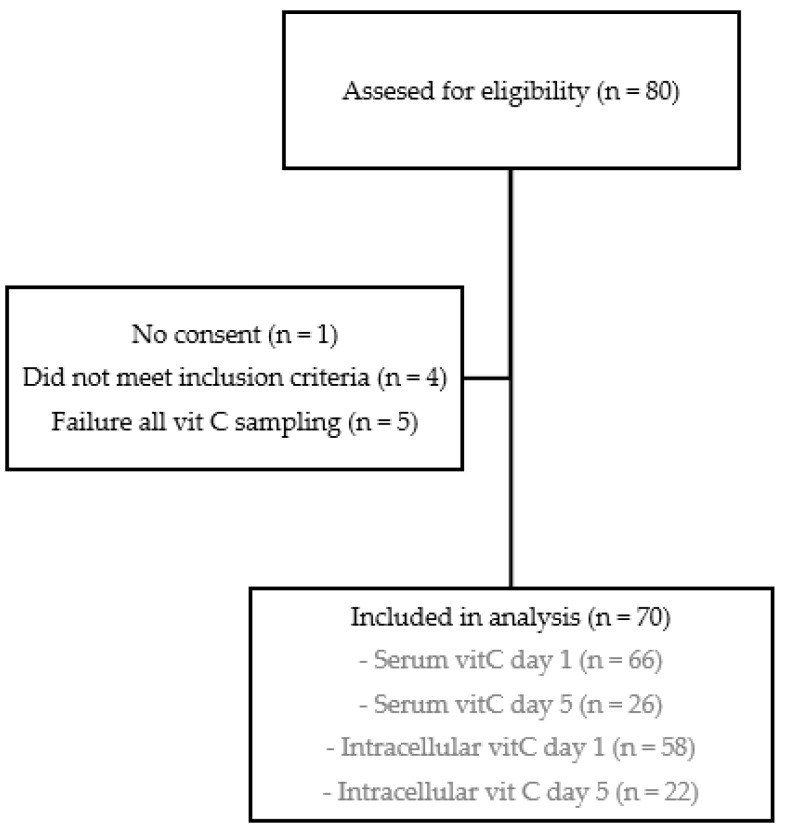
Flowchart of inclusions. Figure explains number of patients assessed for eligibility, patients that were excluded from the study, and finally the patients included in the analysis. Missing data is caused by failed measurements (day 1 serum *n* = 4, day 1 intracellular *n* = 12, day 5 serum *n* = 12, day 5 intracellular *n* = 16), early discharge (day 5 serum *n* = 21, day 5 intracellular *n* = 21), and death (day 5 serum *n* = 1, day 5 intracellular *n* = 1).

A total of 80 patients admitted to the COVID-19 ward at MUMC+ between 2 December 2020, and 23 March 2022, were invited to participate in this study. Four patients did not meet the inclusion criteria, and one patient did not provide informed consent. Additionally, five patients were excluded from the analysis because of failure of all vitamin C measurements (Figure 1). 

### 3.2. Baseline Characteristics

The baseline characteristics of the cohort are summarized in Table 1. The mean age of the patients was 70 ± 11 years, with a male predominance (64%). Almost all patients (except 12) had relevant comorbidities, with cardiac conditions (47%), pulmonary diseases (37%), and diabetes (29%) being the most prevalent. The median duration from the start of symptoms to hospitalization was 7 days. According to the clinical COVID-19 severity classification, 1% were classified as ‘mild’, 15% as ‘moderate’, 60% as ‘severe’, and 27% as ‘critically ill’. A total of five (7%) patients were admitted to the ICU; the other 14 patients were not admitted due to treatment restrictions and received best supportive care. Unfortunately, eleven (16%) patients passed away before being discharged from the hospital. The majority of patients were admitted when the SARS-CoV-2 Alpha variant was dominant (*n* = 56); the other patients were admitted during the Delta wave and the start of the Omicron wave in the Netherlands (*n* = 14).

### 3.3. Serum Vitamin C Levels

**Figure 2:**
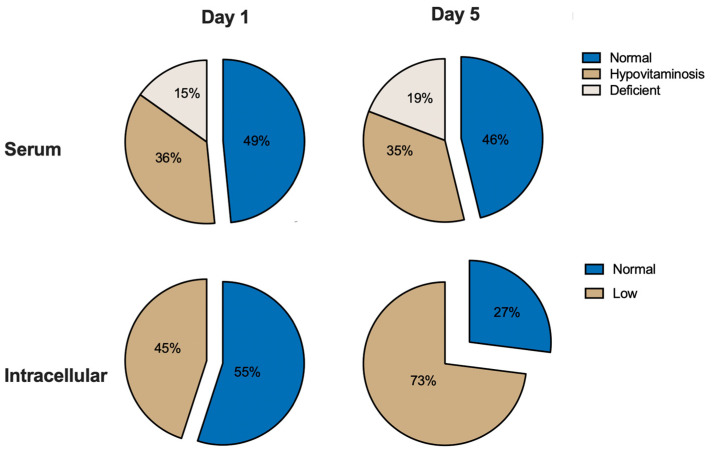
Proportion of normal versus low vitamin C levels in serum and intracellularly on days 1 and 5 of hospitalization.

Upon admission, 49% of patients displayed normal levels of serum vitamin C, while 36% had hypovitaminosis C (<26 µmol/L) and 15% had a vitamin C deficiency (<11 µmol/L). These proportions remained relatively stable, with percentages of 46%, 35%, and 19%, respectively, on day 5 (Figure 2). Optimal vitamin C status (serum/plasma level > 50 µmol/L), as defined by the European Food Safety Authority, is based on the level of vitamin C that is needed to maintain an adequate body pool and ensure proper functioning [37]. It was only present in 11% of the patients on day 1 and in 12% of the patients on day 5. 

Definition of normal vitamin C levels

Serum levels: normal (≥26 µmol/L), hypovitaminosis (<26 µmol/L), deficient (<11 µmol/L).

Intracellular levels: normal (≥4.0 μg/10^8^ cells), low (<4.0 μg/10^8^ cells).

In terms of intracellular vitamin C levels, 55% of the cases on day 1 and 27% of the cases on day 5 of hospitalization had levels above or equal to 4.0 µg/10^8^ cells (10th percentile of the healthy adult population, aged 18–62) previously determined in our center [36] (Figure 2). 

### 3.4. Dynamics of Serum and Intracellular Vitamin C

**Figure 3:**
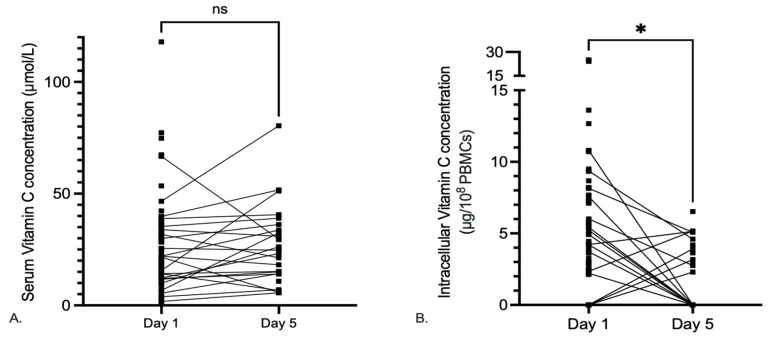
Dynamics of serum and intracellular vitamin C levels during COVID-19 hospitalization. (**A**) Serum vitamin C of patients over time from day 1 (*n* = 70) to day 5 (*n* = 26) of hospitalization, measured by HPLC. Lines connect measurements of the same patients; day 5 measurements were not available for all patients due to early discharge, death, or sampling failure. Complete data (*n* = 26) on days 1 and 5 was compared with a paired *t*-test (*p* = 0.187). (**B**) Intracellular vitamin C over time from day 1 (*n* = 58) to day 5 (*n* = 17) of hospitalization, measured by PBCM isolation, vitamin C extraction, and HPLC. Lines connect measurements of the same patient; day 5 measurements were not available for all patients. Only complete data on day 1 and day 5 (*n* = 17) were compared with a paired *t*-test (*p* = 0.025). ns = non-significant; * = *p* < 0.05.

During hospitalization, the serum vitamin C serum levels did not significantly change from baseline in the majority of the patients between day 1 and day 5 (*p* = 0.187), whereas the intracellular levels significantly decreased between these two time points (*p* = 0.025) (Figure 3). 

### 3.5. Correlation of Serum and Intracellular Vitamin C with COVID-19 Severity

**Figure 4:**
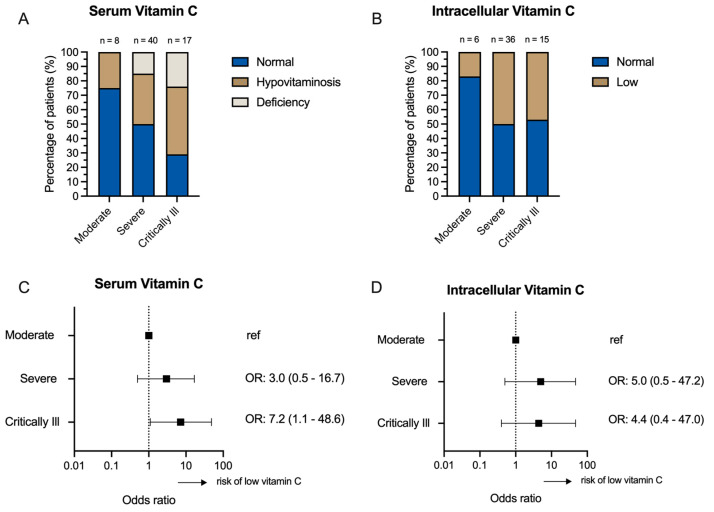
(**A**) Percentage of patients with normal or low serum vitamin C per COVID-19 clinical severity group. (**B**) Percentage of patients with normal or low intracellular vitamin C per COVID-19 clinical severity group. (**C**) Univariate logistic regression analysis of serum vitamin C on day 1 of hospitalization (normal or low *) and clinical COVID-19 severity classification. Mild COVID-19 was not included in the analysis because of the low inclusion number in this category (*n* = 1); therefore, moderate COVID-19 was used as the reference category. Severe (*n* = 40) and critically ill (*n* = 17) COVID-19 were compared to moderate COVID-19 (*n* = 8). (**D**) Univariate logistic regression analysis of intracellular vitamin C on day 1 of hospitalization (normal or low) and clinical COVID-19 severity classification. Mild COVID-19 was not included in the analysis because of the low inclusion number in this category (*n* = 1); therefore, moderate COVID-19 was used as the reference category. Severe (*n* = 36) and critically ill *(n* = 15) COVID-19 were compared to moderate COVID-19 (*n* = 6). * low is defined as hypovitaminosis C or vitamin C deficiency, as described earlier.

A weak but significant negative correlation was observed between the maximal clinical COVID-19 severity classification and serum vitamin C level on the first day of admission (Spearman’s r = −0.341, *p* = 0.005) (Table 2). However, this correlation was no longer present on day 5. There was no correlation between intracellular vitamin C levels and COVID-19 severity at any time point (Table 2). Nevertheless, our data indicate a higher percentage of patients with low serum (Figure 4A) and intracellular (Figure 4B) vitamin C in the group with more severe disease. When comparing the risk of low serum vitamin C levels on day 1 of hospitalization, it was found that critically ill (odds ratio 7.2) COVID-19 patients were more likely to have low serum vitamin C levels compared to COVID-19 patients classified with moderate disease severity; the same trend was observed for severe versus moderate disease severity, though not statistically significant (Figure 4C). A similar trend was observed for intracellular vitamin C levels (Figure 4D). 

Within this cohort, there were 29 patients with an improvement in disease severity between day 1 and day 5 of hospitalization, while 11 patients experienced progressive disease and 30 patients had stable disease (as defined by the clinical COVID-19 severity classification). Median serum vitamin C levels appeared lower in the group with progressive disease when compared to the group with improving disease; however, the ranges were wide, and no statistical differences were observed (Table 3). There was also no statistically significant difference in intracellular vitamin C levels between the progressive and improving disease groups. 

### 3.6. Correlation of Serum and Intracellular Vitamin C with COVID Severity Markers

In this cohort, no correlations were found between serum vitamin C and the SOFA score on both the first and fifth days of hospital admission. Similarly, no correlations were observed between intracellular vitamin C levels and the SOFA score (Table 4). Furthermore, no significant correlations were found between the serum and intracellular vitamin C levels and the duration of the hospital stay or the duration of the intensive care unit (ICU) stay (Table 3). Regarding other biomarkers, only a weak positive correlation was observed between CRP and clinical COVID-19 severity classification, whereas LDH did not show any correlation with COVID-19 severity at all (Table 5). 

## 4. Discussion

In this study, we provide valuable insights into the vitamin C status of hospitalized COVID-19 patients. Our findings demonstrate that a significant proportion of COVID-19 patients display hypovitaminosis C or vitamin C deficiency upon their initial hospital admission. Furthermore, we have observed that serum vitamin C levels remain relatively constant over time. However, we did not investigate the clinical consequences of vitamin C deficiency (e.g., scurvy). These observations align with previously published data on serum vitamin C levels in hospitalized patients suffering from various infections, including COVID-19 [21,38,39,40]. 

Our study introduced a novel approach by investigating not only serum vitamin C but also intracellular vitamin C in PBMCs as a marker for vitamin C storage [11]. Interestingly, when comparing the serum vitamin C status—hypovitaminosis C and vitamin C deficiency—of this COVID-19 cohort with published data from a large British healthy cohort study (36% vs. 12% and 15% vs. 1.4%, respectively) [19,20], the observed percentages in the hospitalized COVID-19 patients were significantly lower (day 1 *p* < 0.001 and day 5 *p* < 0.001, one sample binominal test). This British study is one of the largest studies evaluating vitamin C status in a healthy cohort. A second large study in France showed similar data [41], but smaller studies conducted in Europe and Canada show larger variations in vitamin C levels [20]. In addition, lower vitamin C levels are associated with male sex and increasing age [42]. Since our cohort is predominantly male and the median age is 70 years, the percentage of vitamin C deficiency in the healthy population could be an underestimation. Upon hospital admission, 45% of patients already exhibited low intracellular vitamin C levels, which further deteriorated during hospitalization, resulting in 73% of patients with low levels at day 5. When compared to a healthy population (40 Dutch healthy volunteers with a female predominance (73%) and a mean age of 35 years (range 18–62)), only 10% exhibited this low (below the 10th percentile) intracellular vitamin C level [36] (one sample binomial test: *p* < 0.001 for both days 1 and 5). 

We evaluated intracellular vitamin C levels in PBMCs, including monocytes, NK cells, and T- and B-lymphocytes. These cell types play vital roles in viral clearance, and it is known that SARS-CoV-2 can directly infect these cells, potentially leading to apoptosis [43,44]. Previously, we showed that under normal conditions, all of these immune cells show comparable levels of intracellular vitamin C [36]. We assume that measurement of intracellular vitamin C in the PBMC fraction effectively reflects levels in monocytes and lymphocytes. 

The underlying mechanism behind our observed findings remains unknown. However, it is hypothesized that intracellular vitamin C from PBMCs reflects the body’s vitamin C storage [12]. Our observations suggest that there may be either vitamin C usage or redistribution taking place. Redistribution is the obvious mechanism when serum vitamin C is utilized to scavenge free radicles produced under high oxidative stress caused by the inflammatory response to the SARS-CoV-2 infection. Exhausted serum levels might be replenished by intracellular stores. Another plausible explanation could be the use of vitamin C by the immune cells themselves [10,13]. The role of vitamin C in various immune cells has been described previously [10,13]. Vitamin C is crucial for neutrophil function, T cell development and proliferation, NK cell proliferation, and Th1 and Th17 polarization [13], all of which are important in the clearance of viral invaders like SARS-CoV-2. Data from Carr et al. support the latter hypothesis, as they demonstrated increased vitamin C uptake by neutrophils during infection [33]. If this mechanism also holds true for PBMCs during active SARS-CoV-2 infection, the low intracellular vitamin C levels can be explained by the increased immune cell activation [8,9,10,11,13]. 

Remarkably, we found no strong correlation between serum or intracellular vitamin C levels and the clinical COVID-19 severity classification in this cohort. This lack of correlation could be attributed to the substantial variability in the patient population included in the study. Most patients had pre-existing comorbidities, were on chronic medication, and were aged 65 or older, all of which can potentially influence vitamin C levels but also the severity of COVID-19 (e.g., immunoscenescence in the elderly). This resulted in a diverse patient population at baseline. On the other hand, it is important to note that the cohort did not contain many patients with a clinical COVID-19 severity classification that was ‘mild’ or ‘critically ill’, as we did not allocate patients in each disease severity group. This is because this study was conducted primarily on the COVID-19 ward, and primary inclusions did not take place on the ICU. Additionally, primary care facilities were not included in this study, which was responsible for the lack of patients with mild diseases. The cohort was therefore more homogenous in terms of disease severity than expected. Due to the combination of the heterogeneous patient cohort at baseline and the homogeneous distribution of COVID-19 severity, the study was underpowered to evaluate if vitamin C could be a potential biomarker predicting disease severity. Consequently, the results of our study might be less generalizable than anticipated.

Although the current study did not establish vitamin C levels as definitive biomarkers for assessing COVID-19 severity, we did observe that critically ill patients had higher odds of having low serum vitamin C levels compared to patients with moderate disease severity. Moreover, there was a trend towards lower serum vitamin C levels in patients with progressive disease during the first 5 days of hospitalization. However, it is worth noting that the clinical COVID-19 severity classification used in this study may not be the most accurate instrument for measuring variations in disease severity as it does not effectively discriminate between minor and major changes in the patient’s health status. Our results are in line with findings from previous studies that reported low vitamin C levels in critically ill patients. Low vitamin C has been identified as a risk factor for mortality, though it is influenced by age [38,39,40]. 

Importantly, our study extended beyond analyzing serum vitamin C levels by incorporating intracellular vitamin C analysis, which allowed us to generate hypotheses about a possible underlying mechanism of decreased serum vitamin C levels during infectious diseases. In this cohort, we found no correlation between intracellular and serum vitamin C, which aligns with data from our previous studies in healthy individuals [36]. Although no correlation between intracellular vitamin C levels and COVID-19 severity was observed, we did identify a significant progressive decline in intracellular vitamin C levels in this cohort. This emphasizes the potential importance of maintaining adequate intracellular vitamin C storage, even in critically ill patients. Our study provides a rationale for evaluating both serum and intracellular vitamin C and supports further in vitro and in vivo research to substantiate the role of intracellular vitamin C in disease.

The SOFA score and duration of hospital/ICU stay correlated poorly with serum and intracellular levels of vitamin C in this cohort. Similarly, the commonly used biomarkers, CRP and LDH, showed only a weak correlation and no correlation with COVID-19 severity, respectively. It is important to note that other studies have reported much stronger correlations between these biomarkers and COVID-19 severity [26], again suggesting that the relatively homogenous nature of this cohort in terms of COVID-19 severity may have hindered the detection of strong correlations. Furthermore, it is worth considering that there are numerous other factors known to predispose individuals to low vitamin C levels, such as older age, obesity, smoking status, and diabetes mellitus, amongst others [38,45,46]. Also, in addition to critical illness, hyperglycemia and sepsis are possible causes for decreased vitamin C levels; these were factors that were not evaluated in the current study [7]. These factors may contribute to the complexity of the relationship between vitamin C levels and disease severity observed in this study.

The results from vitamin C intervention trials in COVID-19 patients have yielded conflicting outcomes, as reviewed in [47]. Nevertheless, there appears to be an overall beneficial effect of vitamin C supplementation on severely ill patients, leading to a reduction in early mortality. Conversely, studies focusing on patients with longer-lasting symptoms of COVID-19 have generally not demonstrated a significant effect of vitamin C supplementation [48], which is in line with many interventions utilized in critically ill COVID-19 patients, including convalescent plasma, anti-SARS-CoV-2 antibodies, and antiviral medication. 

In our study, we observed a higher prevalence of vitamin C shortage as the severity of the disease increased, with only a small proportion of patients maintaining optimal vitamin C levels. Optimizing vitamin C status in the early phase of SARS-CoV-2 infection may potentially prevent severe illness and the need for hospital care. The positive effects of early vitamin C supplementation have already been demonstrated in the context of common cold infections [30,49]. However, further research specific to COVID-19 conducted in the primary health care setting would be necessary to confirm this hypothesis.

The limitations of the study are that specific measurements to learn more about the overall vitamin C dynamics were not performed because this study focused on clinical parameters. For example, immune function was not properly evaluated, and no analyses of vitamin C intake or excretion were performed. Additionally, no oxidant levels were measured, though these can highly influence vitamin C levels intracellularly and extracellularly. Lastly, vitamin C’s role as a cofactor for many enzymes and vitamin C’s epigenetic functions were not considered. As there are various enzymes that are assumed to be necessary in infectious disease (e.g., for noradrenalin production and healing), the lack of vitamin C could also partly be explained by this, as well as a poor diet during illness or the high oxidant levels and therefore the high need for scavenging by vitamin C. Nevertheless, we remain curious if these phenomena could also provide an explanation for the persistent deficiency of intracellular vitamin C in PBMCs. In addition, due to loss of follow-up, early discharge, death, or failure of measurements, we have less than 50% of patient data on both days 1 and 5. Valuable data that could have shed more light on the vitamin C metabolism in disease. 

## 5. Conclusions and Future Directions

In conclusion, our study reveals a striking prevalence of hypovitaminosis C, or vitamin C deficiency, among hospitalized COVID-19 patients, emphasizing the need to address this nutritional deficit. Furthermore, our findings suggest that the depletion of intracellular vitamin C storage levels may be attributed to its utilization by immune cells, implicating a vital role in combating infections. Although the correlations between serum vitamin C levels and clinical COVID-19 severity classification appear modest, further investigations within a more heterogeneous group of patients are warranted to better understand this relationship. With the current study, we have generated hypotheses regarding the mechanisms of vitamin C cycling in infectious diseases, opening exciting possibilities for further investigations in in vitro and in vivo studies. The distinct differences observed between serum and intracellular vitamin C warrant further evaluation of both serum and intracellular vitamin C in future observational and interventional studies. Future studies should take into consideration that the physiological role of vitamin C is extensive, and therefore many factors should be addressed in a follow-up study. For a better mechanistic understanding of our findings, we suggest that immune cell and immune function measurements, vitamin C uptake in immune cells, oxidant levels, vitamin C intake and excretion, and possibly also enzymology be evaluated. Additionally, future studies should control for possible confounders such as older age, co-morbidities, and smoking status. Lastly, we suggest that future studies be conducted in a more homogenous group of patients at baseline and that a more even distribution of disease severity be achieved, for example, by allocation at inclusion. The ultimate goal is to identify patients who derive the greatest benefits from targeted vitamin C treatments. By shedding light on the complex dynamics of vitamin C in COVID-19, our study paves the way for advancing clinical strategies aimed at optimizing vitamin C status in patients and improving their overall outcome. 

## Figures and Tables

**Table 1:** Baseline characteristics.

Characteristic	*n* = 70 (Mean ± SD or Median (IQR))
Age (years)	70 ± 12
Gender (*n*, %)	
Male	45 (64%)
Female	25 (36%)
Vitals upon admission	
Oxygen suppletion (L/min)	2 (1–5)
Oxygen saturation (%)	93 ± 3
Comorbidities (*n*, %)	
None	12 (17%)
1	15 (21%)
2	22 (31%)
3+	21 (30%)
Body mass index (BMI)	27.8 (24.4–31.6)
Diabetes mellitus (*n*, %)	20 (29%)
(Hematologic) cancer (*n*, %)	3 (4%)
Medication (*n*,%)	
None	20 (29%)
Immunosuppression	9 (13%)
Anticoagulation therapeutic	14 (20%)
Anticoagulation prophylactic	24 (34%)
Antibiotics	12 (20%)
Chemotherapy	1 (1%)
Abnormal chest X-ray (*n*, %)	40 (57%)
CT-value SARS-CoV-2 PCR	22 ± 6
Time since first symptoms (days)	6.5 (2.3–9.0)
SOFA score	2 (2-3)
Maximal clinical COVID-19 severity classification * (*n*, %)	
Mild	1 (1%)
Moderate	8 (11%)
Severe	42 (60%)
Critically ill (ICU indication)	19 (27%)
Duration of hospital admission (days)	9 (4–14)
Actual ICU admission (*n*, %)	5 (7%)
Duration ICU admission (days)	7 (4–29)
Death (*n*, %)	11 (16%)

Abbreviations: CT: cycle threshold, PCR: polymerase chain reaction, ICU: intensive care unit. * According to Chinese Clinical Guidance for COVID-19 135 Pneumonia Diagnosis and Treatment, 7th edition [34].

**Table 2:** Correlation of serum and intracellular vitamin C levels with maximal clinical COVID-19 severity classification.

	Vitamin C Serum Day 1 (*n* = 66)		Vitamin C Serum Day 5 (*n* = 26)		Vitamin C Intracellular Day 1 (*n* = 58)		Vitamin C Intracellular Day 5 (*n* = 22)	
	*r*(s)	*p*-Value	*r*(s)	*p*-Value	*r*(s)	*p*-Value	*r*(s)	*p*-Value
Maximal clinical COVID-19 severity classification *	−0.341	0.005	−0.169	0.407	−0.082	0.542	−0.142	0.528

Table shows Spearman’s rank correlation of serum and intracellular vitamin C levels with the maximal clinical COVID-19 severity classification during hospitalization. * according to Chinese Clinical Guidance for COVID-19 135 Pneumonia Diagnosis and Treatment, 7th edition [34].

**Table 3:** Mean vitamin C levels in patients with improving progressive COVID-19.

	Improving Disease (*n* = 29)	Progressive Disease (*n* = 11)	*p*-Value
Serum vitamin C day 1	32.1 ± 19.8	19.8 ± 14.4	0.108
Intracellular vitamin C day 1	5.2 ± 6.5	5.4 ± 3.7	0.920

Median serum and intracellular vitamin C levels are shown with interquartile ranges; comparisons were made using unpaired *t*-test, and *p*-values are shown in the table.

**Table 4:** Correlation of serum and intracellular vitamin C with duration of hospital and ICU stay and SOFA score.

	Vitamin C Serum Day 1		Vitamin C Serum Day 5		Vitamin C Intracellular Day 1		Vitamin C Intracellular Day 5	
	*r*(s)	*p*-Value	*r*(s)	*p*-Value	*r*(s)	*p*-Value	*r*(s)	*p*-Value
Duration hospital stay (days)	−0.201	0.105	0.206	0.313	0.008	0.952	−0.032	0.886
Duration of ICU stay (days)	0.029	0.957	-	-	0.143	0.787	-	-
SOFA score	−0.021	0.1	0.156	0.467	−0.017	0.904	−0.240	0.354

Table shows results of Spearman’s rank correlation of serum and intracellular on days 1 and 5 of hospitalization with duration of hospital stay, duration of ICU stay, and SOFA score on day 1 of hospitalization. Analysis for the correlation of vitamin C in serum and intracellularly with duration of ICU stay was not carried out because of the low number of inclusions.

**Table 5:** Correlation of CRP and LDH with clinical COVID-19 severity classification.

	CRP		LDH	
	*r*(s)	*p*-Value	*r*(s)	*p*-Value
Maximal clinical COVID-19 severity classification	0.26	0.030	0.11	0.374

Table shows results of Spearman’s rank correlation of CRP and LDH with duration of maximal clinical COVID-19 severity classification (according to Chinese Clinical Guidance for COVID-19 Pneumonia Diagnosis and Treatment, 7th edition [34]) during hospitalization.

## Data Availability

The data presented in this study are available upon reasonable request from the corresponding author.
